# The lipoprotein NlpD in *Cronobacter sakazakii* responds to acid stress and regulates macrophage resistance and virulence by maintaining membrane integrity: **Running Title**: Identification and characterization of a novel factor involved in acid tolerance and virulence in *Cronobacter sakazakii*

**DOI:** 10.1080/21505594.2020.1870336

**Published:** 2021-01-18

**Authors:** Xuemeng Ji, Ping Lu, Juan Xue, Ning Zhao, Yan Zhang, Lu Dong, Xuejiao Zhang, Ping Li, Yaozhong Hu, Jin Wang, Bowei Zhang, Jingmin Liu, Huan lv, Shuo Wang

**Affiliations:** a Tianjin Key Laboratory of Food Science and Health, School of Medicine, Nankai University, Tianjin, China; b Institute of Radiation Medicine, Chinese Academy of Medical Science and Peking Union Medical Collage, Tianjin, China; c Institute of Infection and Immunity, Taihe Hospital, Hubei University of Medicine, Shiyan, China; d Key Laboratory of Food Nutrition and Safety, Ministry of Education, Tianjin University of Science and Technology, Tianjin, China

**Keywords:** *Cronobacter sakazakii*, lipoprotein, membrane integrity, acid resistance

## Abstract

*Cronobacter sakazakii*, an emerging opportunistic pathogen, is implicated in severe foodborne outbreak infections in premature and full-term infants. Generally, acid tolerance is vital for the pathogenesis of foodborne pathogens; however, its role in *C. sakazakii* virulence remains largely unknown. To screen out acid-tolerance determinants from transposon mutants, anovel counterselection method using gentamicin and acid was developed. Using the counterselection method and growth assay, we screened several acid-sensitive mutants and found that *nlpD* encodes an acid-resistance factor in *C. sakazakii*.

Compared to the wild-type strain, the *nlpD* mutant exhibited attenuated virulence in a rat model. Using macrophage THP-1 cells and a pH probe, we verified that *nlpD* enables bacteria to resist macrophages by resisting acidification. Finally, we confirmed that *nlpD* maintains *C. sakazakii* membrane integrity in acid using propidium iodide permeabilization assays via flow cytometry. Our results confirm that *nlpD* is a novel virulence factor that permits *C. sakazakii* to survive under acid stress conditions. Considering that NlpD is a conserved lipoprotein located in the bacterial outer membrane, NlpD could be used as a target for drug development.

## Introduction

*Cronobacter sakazakii* (formerly *Enterobacter sakazakii*) is an emerging opportunistic foodborne pathogen that causes life-threatening sepsis, meningitis, and necrotizing enterocolitis in premature and full-term infants [1–3]. Since the first report describing an association between *C. sakazakii* infection and consumption of a powdered infant formula (PIF) in Tennessee in 2001 [4], the risk of infection due to the use of PIF in the neonatal health-care setting has been known. Since then, *C. sakazakii* has been widely studied around the world.

The virulence traits and pathogenic mechanisms of *C. sakazakii* remain largely unknown, although initial research has begun to identify specific genes that are involved in the infection process [5–7]. This initial research has revealed that *C. sakazakii* is able to form biofilms [8], possesses iron acquisition genes [9], and persists in human macrophages [10].

Acid resistance and desiccation resistance are very important for *C. sakazakii* infection; extraordinary desiccation resistance enables bacteria to survive in PIF, which is the main vehicle of *C. sakazakii* infection [8]. In addition, *C. sakazakii* can adapt to a low-pH environment [11]. This characteristic may be associated with the fact that the organism encounters an acidic environment during its passage through the stomach and during its survival within macrophages.

The gastric pH of infants varies from 2.9 to 5.8 after feeding [11,12]. In addition, it has been reported that some organic acids present in the stomach may have antibacterial effects [13]. *C. sakazakii* can grow at pH values as low as 4.1 [14] and has been shown to tolerate exposure to low pH for long periods [15]. The uptake of invading microorganisms by macrophages is an intrinsic defense response used to kill harmful bacteria. Phagocytes engulf bacteria and form phagolysosomes. The phagolysosomes fuse with lysosomes containing acid hydrolases and free radicals that kill phagocytized bacteria. It has also been reported that acidification may play an important role in killing bacteria [16,17]. *C. sakazakii* has been shown to survive and even to multiply within macrophages [10,18].

Studies have shown that the acid determinants of bacteria also affect bacterial virulence. For example, deletion of the acid-tolerance response gene *dsrA* of *Salmonella enterica* results in defective invasion efficacy and renders the bacterium unable to cause gut inflammation in mice [19]. In addition, the *yaeB* mutation in *Salmonella typhimurium* results in increased acid sensitivity and decreased survival within macrophages [20]. Moreover, acid-adapted bacteria demonstrate increased virulence. For example, compared to the nonacid-adapted wild-type *Listeria monocytogenes*, the acid-adapted strain and a constitutively acid-tolerant mutant showed more invasion of enterocyte-like cells and greater survival within activated macrophages [21]. However, little information is available on the association between acid tolerance and virulence in *C. sakazakii*.

The objective of this study was to investigate genes putatively associated with acid tolerance-mediated resistance to macrophages and with pathogenicity. We found that *nlpD* rendered *C. sakazakii* resistant to acid stress in the stomach and within macrophages by maintaining the bacterial pH. NlpD is a novel virulence factor of *C. sakazakii* and could be a drug target in the future.

## Results

### Screening for acid-resistance genes of *C. sakazakii*

Transposon mutagenesis can efficiently produce a large number of near-random mutations of the bacterial genome; however, subsequent selection of stress-sensitive mutants generally requires a large number of single colony screenings since the rate of transposon insertion is quite low. Here, we developed a counterselection method that relies on the antagonism of an acidic environment to bactericidal antibiotics that specifically kill actively replicating bacteria. Gentamicin is a quick-acting bactericidal antibiotic that is most efficient against rapidly growing bacteria [22]. Therefore, exposure to a moderately acidic pH that inhibits the growth of acid-sensitive mutants should protect these strains from the bactericidal effect of gentamicin, while wild-type strains and mutants that are able to grow under moderately acidic conditions should be killed quickly. Three concentrations of gentamicin were tested for bactericidal activity against *C. sakazakii* (Fig S1A). Strong bactericidal activity was observed at all three gentamicin concentrations. At 200 mg/L, gentamicin reduced the number of viable *C. sakazakii* to below the detection level within 60 min. To further select a suitable acidic pH, an LB medium adjusted to various pH values using hydrochloric acid was investigated for its inhibitory effects on the growth of *C. sakazakii*. The *dnaK* deletion mutant of *C. sakazakii* was generated as an acid-sensitive control. Compared to neutral pH, pH 3.0 showed a significant inhibitory effect on the growth of *ΔdnaK C. sakazakii* compared to its effect on the growth of wild-type *C. sakazakii* (WT) after culture for 60 min (Fig S1B).

In order to establish a counterselection method that depends on the antagonistic effects of acidic pH on the bactericidal activity of gentamicin, the survival of *C. sakazakii* was studied after exposure to a combination of acidic pH and gentamicin. The acid-sensitive *ΔdnaK* mutant strain was capable of withstanding the killing of gentamicin after growth inhibition by acidic pH (Fig S1C). By contrast, rapid killing of the acid-resistant wild-type strain due to gentamicin was observed; the number of viable bacteria of the resistant strain was below the detection threshold after 60 min. Therefore, it appears that the combination of an acidic pH and gentamicin has the desired counterselective effect on acid-sensitive strains of *C. sakazakii*.

**Figure 1.**
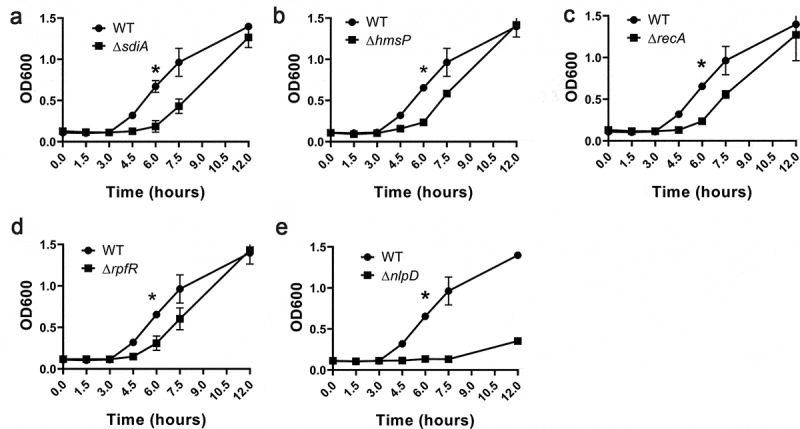
Phenotype verification of five acid determinants using markerless deletion mutants

Using this method, we screened several acid-sensitive mutants and identified the insertion sites of their mutations. The phenotype of acid sensitivity was further verified by knocking out the open reading frames of the genes. Finally, we identified five genes, *sidA, hmsP, recA, rpfR,* and *nlpD*, that enable *C. sakazakii* to resist acidic environments (Figure 1) but that do not affect the growth rate of the bacterium in neutral medium (data not shown). NlpD, an extracellular membrane protein, is thought to be related to biofilm formation [23], but it has not previously been reported to be related to acid tolerance. Therefore, the role of *nlpD* in the acid tolerance and virulence of *C. sakazakii* was investigated in this study.

Growth of (A) wild-type (WT) and Δ*sdiA C. sakazakii*, (B) WT and Δ*hmsP C. sakazakii*, (C) WT and Δ*recA C. sakazakii*, (D) WT and Δ*rpfR C. sakazakii* and (E) WT and Δ*nlpD C. sakazakii* in LB medium at pH 3.0. To initiate experiments, *C. sakazakii* strains were cultured in Luria-Bertani (LB) overnight at 37°C. In all cases, the estimated initial bacterial densities were 0.1 optical density at 600 nm (OD600). Optical densities of *C. sakazakii* cultured in LB medium at pH 3.0 were measured immediately before (time zero) and at the indicated time points. Each data point represents the average and standard deviation of three biological repeats.

### *nlpD* knockout reduces bacterial virulence

During the process of *C. sakazakii* infection, the pathogen is exposed to a variety of acidic environments, including the gastric acid environment, the intestinal acid environment, and the acidic environment inside macrophage phagocytic vesicles. We have verified that *nlpD* contributes to acid stress induced by hydrochloric acid, which is the main component of gastric acid. However, whether *nlpD* acts similar under organic acid stress that exists in intestinal and macrophage remains unknown. Therefore, the survival of wildtype, nlpD mutant, and complement strains was tested at low pH adjusted by a diversity of organic acids. Compared with wild-type strain, knockout showed significant growth disadvantages in medium acidified regardless of the pH regulated by acetic acid, butyric acid, or lactic acid (figure S2). These results clearly demonstrated that *nlpD* confers *C. sakazakii* with resistance to both inorganic acid stress and organic acid stress.

**Figure 2.**
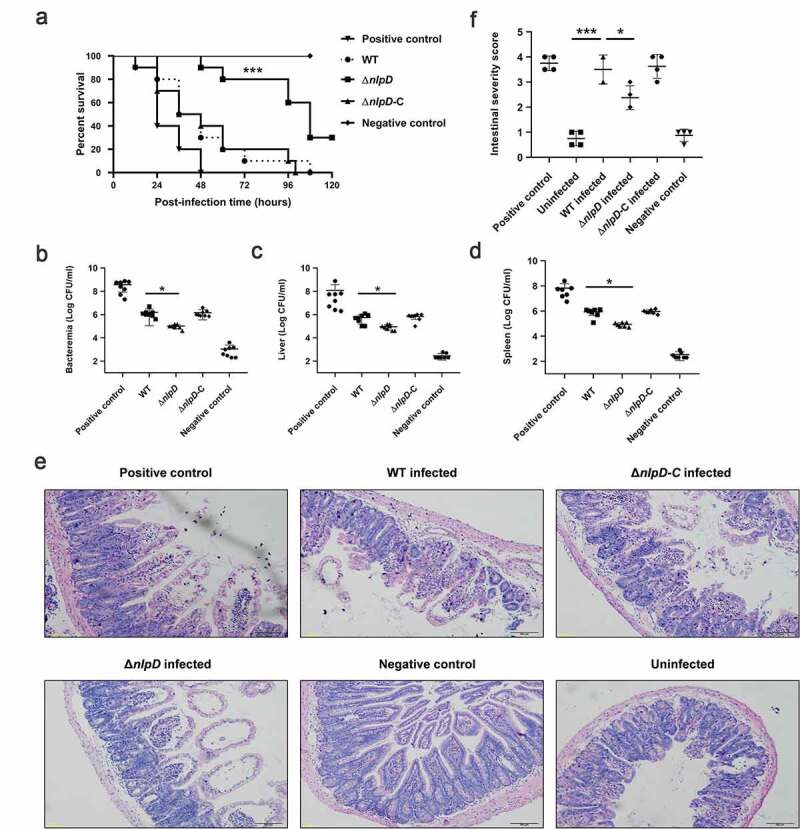
The acid determinant *nlpD* is involved in the pathogenicity of *Cronobacter sakazakii.*

As an acid-resistance factor, *nlpD* may affect the pathogenicity of *C. sakazakii*. Three groups of 3-d-old rats were orally infected with WT, *ΔnlpD,* and *ΔnlpD* complemented strains of *C. sakazakii*, and the survival of the animals was monitored for more than 5 d. Rats infected with WT bacteria or with strains in which the mutations were complemented began to die at 12 hours, and 80% of the animals had died by 72 hours. In contrast, rats infected with *ΔnlpD* bacteria began to die at 48 hours, and 80% of them survived for at least 72 hours after infection. The significant difference between the *ΔnlpD* and WT strains was identified by a survival curve (Figure 2a). Whether *nlpD* affects the colonization of *C. sakazakii* in mice was also investigated. The bacterial load of *ΔnlpD* bacteria in blood (Figure 2b), liver (Figure 2c), and spleen (Figure 2d) was 8- to 20-fold lower than that of WT bacteria. In addition, intestinal tissue from rats infected with the WT strain showed dilation and necrosis by 48 h post-infection, accompanied by perforation and destruction of villi, whereas intestinal tissue damage was significantly less severe in *ΔnlpD*-infected rats (Figure 2e and figure 2f). These results indicate that *nlpD* knockout decreases the virulence of *C. sakazakii*.

(A) Survival of rat pups during the 120-hour period following oral infection with various bacterial strains. Combined data from three independent experiments are shown (log-rank test; ***, P < 0.001). (B–D) Colony-forming units (CFU) present in the blood, liver, and spleen of infected rats at 24 hours post-infection. Blood was collected from the facial vein, and the liver and spleen were harvested and homogenized in ice-cold PBS for use in colony enumeration. (E) Histopathologic examination of intestinal tissues of uninfected, WT-infected, and *ΔnlpD*-infected rats. Scale bars, 100 μm. (F) Intestinal sections obtained at 24 hours post-infection were graded as grade 0 (normal) to grade 4 (severe) by a pathologist blinded to the groups according to morphological changes (n = 4). *Salmonella enterica* serovar Typhimurium and *E. coli* DH5α serve as positive and negative controls, respectively.

### *nlpD* does not affect bacterial adhesion

**Figure 3.**
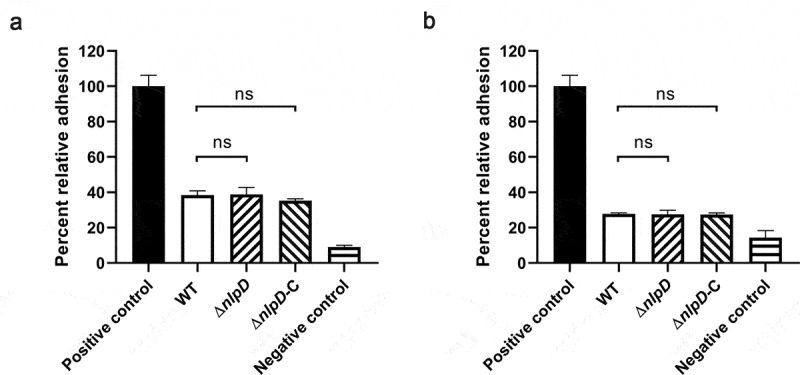
*nlpD* is not involved in adherence

NlpD is a lipoprotein that is thought to be involved in biofilm formation [24]. We next tested the effect of *nlpD* on bacterial adhesion. A gentamicin protection test was conducted to study the role of *nlpD* in colonization by *C. sakazakii* of the small intestinal epithelial cell line Caco-2 and the human brain microvascular endothelial cell line HBMEC. No significant differences in colonization by WT, Δ*nlpD,* or Δ*nlpD*-C *C. sakazakii* were observed in either of these cell lines (Figure 3). Therefore, it is clear that *nlpD* does not affect colonization by *C. sakazakii in vitro*.

(A, B) Relative adhesion of WT, Δ*nlpD* mutant, and complemented strain to Caco-2 (A) and human brain microvascular endothelial cells (HBMECs) (B). The data are presented as the mean and standard deviation of three biological repeats; n = 3 (Student’s t-test; ns, no statistically significant difference) of three biological repeats. To initiate experiments, *C. sakazakii Salmonella enterica* serovar Typhimurium and DH5α were cultured in LB overnight at 37°C. *Listeria monocytogenes* EGD-e was cultured in BHI overnight at 37°C. *Salmonella enterica* serovar Typhimurium was included as a positive control in adhesion to Caco-2 (A). Positive control in adhesion to HBMECs was *Listeria monocytogenes* EGD-e (B). *E. coli* DH5α served as a negative control in adhesion to both Caco-2 and HBMECs.

### *C. sakazakii* requires nlpD for survival in macrophages

**Figure 4.**
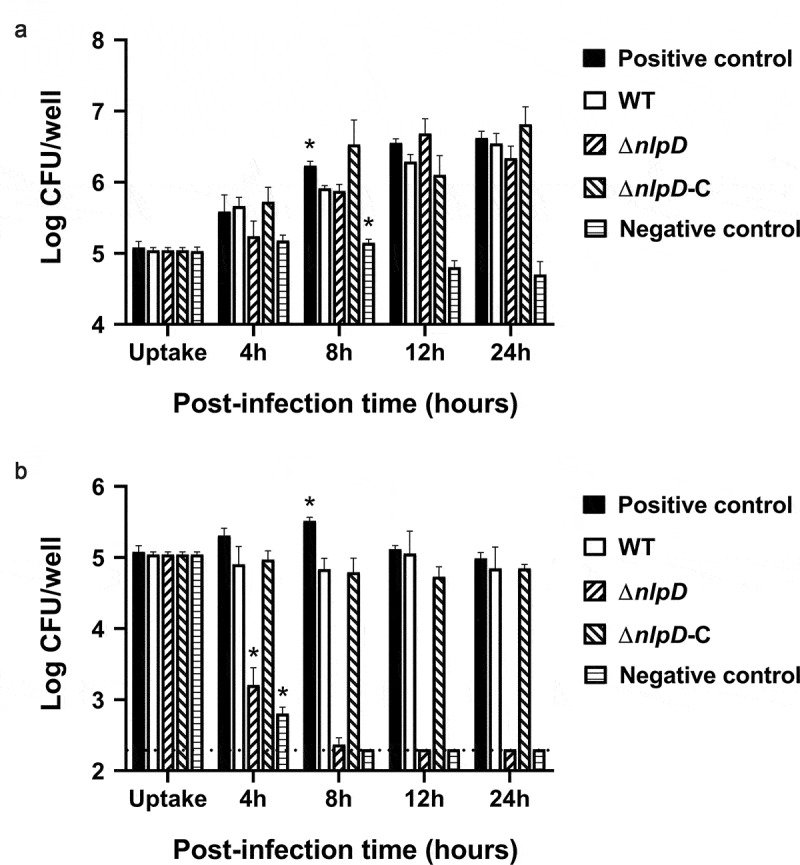
The Δ*nlpD* mutant shows attenuated survival within activated macrophages

Previous studies have found that *C. sakazakii* resists killing by macrophages [18,25]. Phagocytes mainly use acidic environments, ROS, and lysozyme to kill intracellular bacteria. Based on our discovery that *nlpD* is an acid-resistance factor in *C. sakazakii, nlpD* may be involved in the tolerance of this bacterium to killing by macrophages. To determine whether *nlpD* affects the macrophage tolerance of *C. sakazakii*, non-activated and IFN-γ-activated macrophages derived from human THP-1 cell lines were challenged with WT, Δ*nlpD,* or Δ*nlpD*-C strains. All of the strains survived and replicated in non-activated macrophages (Figure 4a). In IFN-γ-activated macrophages, the CFU of the WT and *nlpD*-C strains remained basically unchanged after the addition of gentamicin (Figure 4b); however, the number of viable Δ*nlpD* bacteria showed a continuous decline after the addition of gentamicin, and after 8 hours, the CFU of Δ*nlpD* bacteria was below the detection threshold. Thus, the Δ*nlpD* strain did not resist killing by macrophages; therefore, *C. sakazakii* needs *nlpD* to survive in macrophages.

(A, B) Survival of WT, Δ*nlpD*, and complemented strains within nonactivated macrophages (A) and IFN-γ-activated macrophages. (B) The cells were harvested and resuspended in 1% Triton X-100 buffer to separate aggregates of bacteria within the cells at the indicated time points. THP-1 cells were differentiated into macrophages using phorbol 12-myristate 13-acetate treatment at 60 ng/mL for 48 hours, and interferon-γ (IFN-γ) was used to activate macrophages for enhanced microbial killing. *C. sakazakii Salmonella enterica* serovar Typhimurium and DH5α were cultured in LB overnight at 37°C. Strains were added to THP-1 cells at an MOI of 100. The bacteria were plated on LB agar, and CFU was counted after culture for 24 hours. The assays were repeated three times biologically, and the data represent the average and standard deviation. Salmonella enterica serovar Typhimurium and DH5α served as positive and negative controls, respectively. *Comparison of indicated strain with WT yielded p < .05 with one-way ANOVA.

### *Cronobacter sakazakii* requires *nlpD* to maintain its pH in macrophages

**Figure 5.**
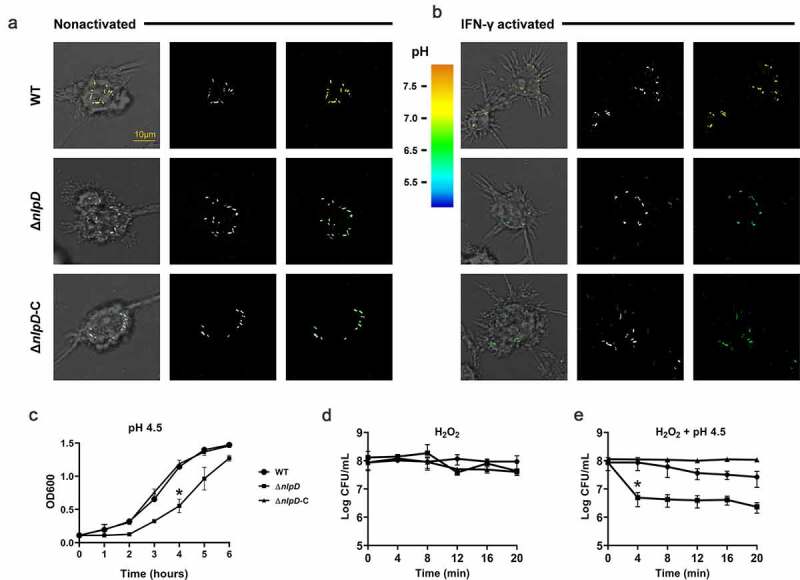
*nlpD* deletion attenuates bacterial survival within IFN-γ-activated macrophages

To determine whether the reduced macrophage survival of *nlpD* was due to the acidic environment inside the macrophage, the *pHluorin2* gene, which encodes an enhanced, ratiometric, pH-sensitive green fluorescent protein, was cloned into the pUC57 vector and transformed into *C. sakazakii*. Fluorescence images were generated using a confocal microscope, and the pH of the bacterial cells was analyzed. In IFN-γ-activated macrophages, the pH of the WT strain was observed to be above 6, while that of the Δ*nlpD* strain was below 5 (Figure 5a and 5b). Therefore, the presence of *nlpD* maintains the internal pH of the bacteria in macrophages. However, it is surprising that a pH equal to that within macrophages does not kill the Δ*nlpD* strain. Δ*nlpD* still grew at a pH of 4.5, similar to the pH within lysosomes, although it showed slower growth than the WT strain (Figure 5c). Therefore, the hypothesis that the synergistic action of low pH and other factors present in macrophages can kill Δ*nlpD* was proposed. The synergistic effect of pH 4.5 and ROS was verified by the marked reduction in the *in vitro* survival of the Δ*nlpD* mutant in H_2_O_2_ at pH 4.5 (Figure 5e); this reduction in survival did not occur when the mutant was exposed to H_2_O_2_ at a neutral pH (Figure 5d). These results clearly indicate that *nlpD* indeed facilitates resistance to the synergistic effect of bactericidal acidity and reactive oxygen species in macrophages.

Transmitted images with overlays of bacteria in green (left), fluorescent bacteria (center), and pseudocolor images of the 410:470 excitation ratio (right) of nonactivated (A) and IFN-γ–activated (B) macrophages 24 h after infection with WT, the Δ*nlpD* mutant, and the complemented mutant are shown. Scale bar, 10 μm. (C) Sensitivities of *C. sakazakii* strains to pH 4.5. (D) Sensitivities of *C. sakazakii* strains to 200 mM hydrogen peroxide (H_2_O_2_). (E) Sensitivities of *C. sakazakii* strains to a combination of 200 mM hydrogen peroxide (H_2_O_2_) and pH 4.5. To initiate experiments, *C. sakazakii* strains were cultured in Luria-Bertani (LB) overnight at 37°C. In all cases, the estimated initial bacterial densities were 0.1 optical density at 600 nm. Optical densities or CFU counts of *C. sakazakii* were determined immediately before (time zero) and at the indicated time points. All data represent the mean and standard deviation of three biological repeats.

### *nlpD* activation depends on EnvZ

**Figure 6.**
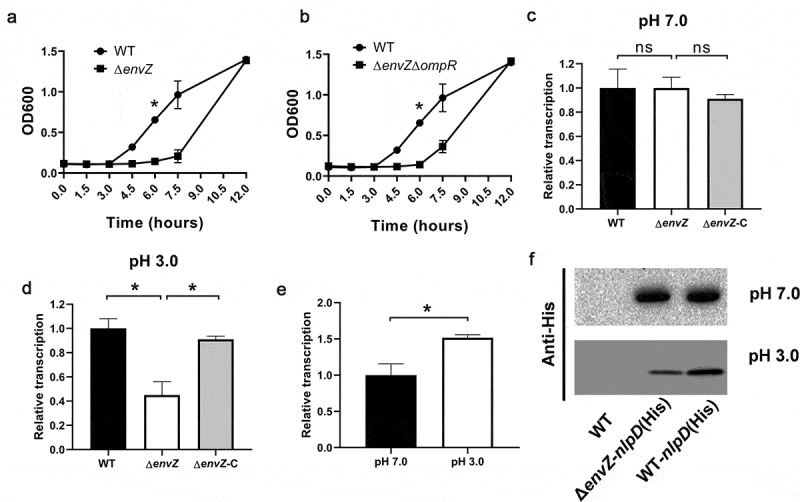
Reduced *nlpD* expression in the Δ*evnZ* mutant

The EnvZ/OmpR two‐component system has been reported to be involved in signal transduction during acid stress [26,27]. In *C. sakazakii*, we also found that Δ*evnZ* and Δ*evnZ*Δ*ompR* showed attenuated acid resistance compared to WT (Figure 6a and 6b). Considering that NlpD is a lipoprotein located in the outer membrane and that the two-component system can widely activate the expression of outer membrane proteins [28–30], we speculated that the EnvZ/OmpR two‐component system might affect the expression of *nlpD*. To determine whether the absence of EnvZ affects the expression of *nlpD*, RT-qPCR was used to measure the level of transcription of *nlpD* in the WT and Δ*evnZ* strains. In neutral medium, Δ*evnZ* showed a similar level of *nlpD* transcription compared to the WT strain (Figure 6c). In the acidic environment, a significant reduction in *nlpD* transcription in Δ*evnZ* was observed compared with the WT strain (Figure 6d), suggesting that *nlpD* transcription is regulated by EnvZ. At the same time, the results showed that *nlpD* transcription was significantly higher in an acidic pH environment than at neutral pH, suggesting that *nlpD* transcription is activated by acidic environments (Figure 6e). In addition, western blotting was used to directly detect the expression of His6-tagged NlpD. The NlpD level in Δ*evnZ* was significantly lower than that in the WT strain under acidic conditions, but no difference was observed at neutral pH (figure 6f). These results indicate that the activation of *nlpD* in acidic environments is dependent on the presence of EnvZ.

(A, B) Growth curves of wild-type (WT), Δ*envZ* (A), *and* Δ*envZ*Δ*ompR* (B) strains in LB medium at pH 3.0. To initiate experiments, *C. sakazakii* strains were cultured in Luria-Bertani (LB) overnight at 37°C. In all cases, the estimated initial bacterial densities were 0.1 optical density at 600 nm. Optical densities of *C. sakazakii* were determined immediately before (time zero) and at the indicated time points. (C, D) Relative transcription of *nlpD* in WT, Δ*evnZ,* and the Δ*evnZ* complemented strain at neutral pH (C) and at pH 3.0 (D). (E) Relative transcription of *nlpD* in the WT strain at neutral pH and at pH 3.0. (F) Western blotting analysis of the levels of His6-tagged NlpD in WT, Δ*evnZ-nlpD* (His), and WT*-nlpD* (His) at neutral pH and at pH 3.0. All data represent the average and standard deviation of three biological repeats performed.

### *nlpD* maintains membrane integrity in acid

Next, we explored the molecular mechanisms responsible for the function of *nlpD* that were observed above. We have disclosed that in addition to *nlpD*, four other genes *rpfR, hmsP, sdiA,* and *recA* also conferred *C. sakazakii* acid resistance (Figure 1). Moreover, acid environment could promote the transcription of *hmsP, sdiA,* and *recA* in *C. sakazakii* (Fig S3 B-D). However, the transcription of *rpfR, hmsP, sdiA,* and *recA* was hardly changed in *nlpD*-knockouted strain in neither neutral pH nor pH 3.0 (Fig S3), suggesting *nlpD* provides acid resistance but independent of *rpfR, hmsP, sdiA,* and *recA*.

**Figure 7.**
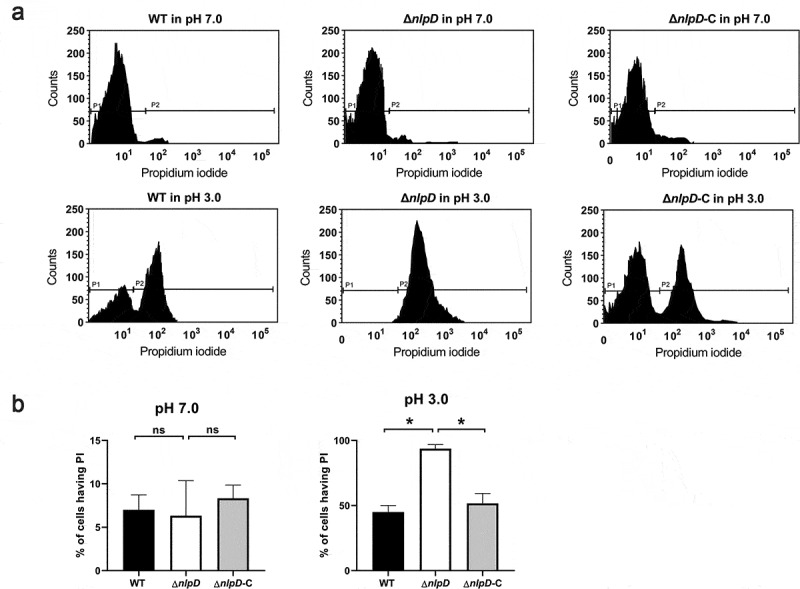
Membrane permeabilization of *C. sakazakii* by PI uptake assay

A low-pH environment can affect the function of proteins, including the proteins of intermediate metabolism and the bacterial envelope. Previous studies have shown that a low pH can affect the function of the bacterial membrane, including increasing the sensitivity of bacteria to hydrophobic drugs, the loss of mobility, and reducing the ability to form biofilm [31], suggesting that a low pH can cause bacterial membrane damage. As a membrane protein, we speculate that *nlpD* could maintain the membrane integrity of *C. sakazakii* at a low pH. Consistent with this conjecture, we observed an increase in the permeability of propidium iodide to *C. sakazakii* at pH 3.0 compared with pH 7.0 (Figure 7a). However, the *nlpD* mutant significantly increased the permeability of propidium iodide in an acidic environment (Figure 7b), suggesting that *nlpD* could maintain the integrity of the bacterial cell membrane in an acidic environment.

(A) Membrane permeabilization of *C. sakazakii* at pH 7.0 or pH 3.0. Logarithmic *C. sakazakii* BAA894 and its Δ*nlpD* mutant were analyzed for membrane permeabilization after incubation in pH 3.0 or pH 7.0 for 2 hours. A total of 10,000 cells were acquired for each flow cytometry analysis. (B) Histograms showing the percent of cells having PI based on data obtained by flow cytometry assays described in Figure 7a of three independent experiments. These data represent the mean (±SD) of three independent experiments (*P ≤ 0.05).

## Discussion

The low pH of gastric secretions is considered the body’s first line of defense against food-borne pathogens. The ability of bacteria to resist being killed by acid during passage through the stomach increases their likelihood of colonizing the intestines and causing an infection [32]. Thus, foodborne pathogens have evolved a variety of acid-tolerance genes for survival in these acidic environments [33–36]. Although many studies have identified a series of acid determinants *in vitro*, only a few recent reports have linked bacterial acid-tolerance genes to bacterial virulence. In our work, we found that the acid-tolerance gene *nlpd* of *C. sakazakii* confers tolerance to macrophages and is a virulence factor. Our study suggests that more attention should be paid to the search for virulence factors from the perspective of acid-tolerance genes.

Our study demonstrated for the first time that *C. sakazakii* can grow at pH 3.0, while previous investigations have found that *C. sakazakii* can withstand an acidity of pH 3.5 [37]. In fact, *C. sakazakii* exists widely in the environment and in food, where the environmental pH changes over time [38]. Therefore, it is not surprising that *C. sakazakii* has extraordinary acid tolerance.

NlpD was found to confer macrophage tolerance as well as tolerance of low pH. Previous studies have found that macrophage removal aggravates *C. sakazakii* infection [25], suggesting that macrophages provide protection against *C. sakazakii* infection in mice. Moreover, we found that macrophages can kill *nlpD*-knockout bacteria efficiently, indicating that these cells do indeed play a bactericidal role, but *nlpD* provides macrophage tolerance. Therefore, the macrophage tolerance of *C. sakazakii* has an important impact on the pathogenicity of the bacterium.

Macrophages rely on acidity, ROS, and other substances to kill intracellular bacteria [39]. Bacteria have evolved multiple mechanisms to resist killing by macrophages. For example, the molecular chaperone DnaK can repair proteins that have been damaged by macrophages and contributes to the survival of *Salmonella* within macrophages [40]. The efflux pump EmrKY contributes to the survival of *Shigella* within macrophages [41]. In addition, a family of surface-exposed virulence factors termed “macrophage infectivity potentiators” (MIPs) have been described in intracellular microorganisms, and these virulence factors are necessary for the survival of *Neisseria gonorrhoeae* [42] and *Legionella pneumophila* [43] within macrophages. Recently, the *mip*-like gene *fkpA* of *C. sakazakii* has been shown to play a role in macrophage resistance, but its role in virulence has not been elucidated [10]. Previous studies have mostly considered that *nlpD* is related to envelop integrity and iron acquisition [44–46]. However, a few studies reported that *nlpD* affects virulence in the phytopathogens *Xanthomonas* [47] and *Yersinia pestis* [48]. Except in *Yersinia pestis*, however, the virulence mechanism of *nlpD* remains poorly understood. Our results reveal for the first time the relationship between *nlpD* and acid resistance and demonstrate that *nlpD* leads to macrophage tolerance and virulence based on an acid-tolerance mechanism.

Similar to the observation in *Vibrio cholerae, Salmonella dublin*, and *Escherichia coli, rpoS* in *C. sakazakii* was identified to locate downstream of *nlpD* as well. *rpoS* has been verified to be associated with the resistance to various environmental stresses, such as oxidative stress, carbon starvation, UV irradiation, and acidic conditions. The adjacent location of *nlpD* and *rpoS* clusters potentially indicated the synergistic effect on environmental stress responses. However, the stop codon introduced by the point mutation C994A in *rpoS* of *C. sakazakii* leads to a premature termination of the sequential translation. The defect of *rpoS* in *C. sakazakii* could potentially pose an emphatic participation of *nlpD* in bacterial acid resistance and pathogenicity.

Two-component systems are widely used by bacteria to sense and respond to environmental changes. We found that *C. sakazakii* can regulate the expression of the acid stress gene *nlpD* through the EnvZ-OmpR two-component system. Similarly, the acid response in Group B Streptococci is regulated by the CovS/CovR two-component system [49]. In fact, EnvZ-OmpR is a two-component system that exists widely in bacteria and is believed to be related to biofilm formation and osmotic tolerance [50–52]. EnvZ has also been widely reported to regulate the expression of a variety of outer membrane proteins [53–55]. Our experiments demonstrate that EnvZ can sense external acidic environments and can regulate the level of the outer membrane protein NlpD, thereby affecting the acidic tolerance of bacteria.

NlpD is a conserved protein present in bacteria that plays an important role in pathogenicity and may serve as a target for future drug development.

## Materials and methods

### Bacterial strains, plasmids, and growth conditions

The strains and plasmids used in this study are listed in Table 1, and the primers are listed in Table 2. Bacteria were stored in LB broth containing 15% glycerol (Biosharp, China) at −80°C. To initiate all experiments, strains were revived in LB broth (Oxoid, UK). When necessary, antibiotics were added at final concentrations of 100 μg mL^−1^ ampicillin (Sangon, China) or 50 μg mL^−1^ kanamycin (Sangon, China). *E. coli* DH5α (Weidi, China) was used as the host for the preparation of plasmid DNA, and *E. coli* S17 lambda pir (Weidi, China) was used for preparation of the pCVD442 suicide vector [56]. Plasmid construction was performed according to standard protocols with the minor modification that cloning of PCR fragment into the linearized vector was accomplished using a commercial seamless cloning and assembly kit (Vazyme, China). pCVD442 (Miaolingbio, China) was linearized by PCR using the primer pair pCVD442-fwd and pCVD442-rev, and the upstream and downstream fragments of genes to be knocked out were amplified by PCR using the primers listed in Table 2. In-frame deletion mutants were generated using the pCVD442 suicide vector method described previously [57]. Deletion mutants were complemented using the low-copy vector pACYC184 (Miaolingbio, China). Δ*nlpD* complementation was performed using the pACYC184 plasmid containing an *nlpD* gene fragment amplified using the primer pair *nlpD*-comp-fwd and *nlpD*-comp-rev. *nlpD*(his) complementation in the Δ*nlpD* mutant was performed using the pACYC184 plasmid containing an *nlpD*(his) gene fragment amplified using the primer pair *nlpD*(his)-fwd and *nlpD*(his)-rev. Transformation and selection of *C. sakazakii* were performed using the method described previously.

**Table 1.** Bacterial strains and plasmids used in this study

Strains, plasmids	Description	Reference, source
*Cronobacter sakazakii*		
WT	Wild-type *Cronobacter sakazakii* BAA-894	[64]
Δ*dnaK*	Markerless deletion mutant Δ*dnaK*	This study
Δ*sidA*	Markerless deletion mutant Δ*sidA*	This study
Δ*hmsP*	Markerless deletion mutant Δ*hmsP*	This study
Δ*recA*	Markerless deletion mutant Δ*recA*	This study
Δ*rpfR*	Markerless deletion mutant Δ*rpfR*	This study
Δ*nlpD*	Markerless deletion mutant Δ*nlpD*	This study
Δ*envZ*	Markerless deletion mutant Δ*envZ*	This study
Δ*envZ*Δ*ompR*	Markerless deletion mutant Δ*envZ*Δ*ompR*	This study
Δ*nlpD*-C	*nlpD* complementation in Δ*nlpD*	This study
Δ*nlpD-nlpD*(his)	*nlpD*(his) complementation in Δ*nlpD*	This study
Δ*envZ-nlpD*(his)	*nlpD*(his) complementation in Δ*envZ*	This study
WT*-nlpD*(his)	*nlpD*(his) complementation in WT	This study
WT-*pHluorin2*	WT harboring pACYC184-*pHluorin2*	This study
Δ*nlpD-pHluorin2*	Δ*nlpD* harboring pACYC184-*pHluorin2*	This study
Δ*nlpD*-C-*pHluorin2*	Δ*nlpD*-C harboring pACYC184-*pHluorin2*	This study
*Escherichia coli*		
*E. coli* DH5α	Strain for construction and controls	[22]
S17 lambda pir	Strain for construction harboring lambda pir	[65]
S17 lambda pir-Δ*dnaK*	S17 lambda pir harboring pCVD442-Δ*dnaK*	This study
S17 lambda pir-Δ*sidA*	S17 lambda pir harboring pCVD442-Δ*sidA*	This study
S17 lambda pir-Δ*sidA*	S17 lambda pir harboring pCVD442-Δ*sidA*	This study
S17 lambda pir-Δ*sidA*	S17 lambda pir harboring pCVD442-Δ*sidA*	This study
S17 lambda pir-Δ*rpfR*	S17 lambda pir harboring pCVD442-Δ*rpfR*	This study
S17 lambda pir-Δ*nlpD*	S17 lambda pir harboring pCVD442-Δ*nlpD*	This study
S17 lambda pir-Δ*envZ*	S17 lambda pir harboring pCVD442-Δ*envZ*	This study
S17 lambda pir-Δ*envZ*Δ*ompR*	S17 lambda pir harboring pCVD442-Δ*envZ*Δ*ompR*	This study
*Salmonella enterica*		
Salmonella enterica serovar Typhimurium	Standard/reference strain used as control	[66]
*Listeria monocytogenes*		
*Listeria monocytogenes* EGD-e	Standard/reference strain used as control	[67]
Plasmids		
pACYC184	Low-copy plasmid	[64]
pACYC184-*nlpD*	*nlpD* complementation vector	This study
pACYC184-*nlpD*(his)	vector harboring *nlpD* fused with His tag	This study
pACYC184-*pHluorin2*	pACYC184 harboring *pHluorin2*	This study
pCVD442	Suicide plasmid for markerless deletion	[68]
pCVD442-Δ*dnaK*	*dnaK* deletion plasmid	This study
pCVD442-Δ*sidA*	*sidA* deletion plasmid	This study
pCVD442-Δ*hmsP*	*hmsP* deletion plasmid	This study
pCVD442-Δ*recA*	*recA* deletion plasmid	This study
pCVD442-Δ*rpfR*	*rpfR* deletion plasmid	This study
pCVD442-Δ*nlpD*	*nlpD* deletion plasmid	This study
pCVD442-Δ*envZ*	*envZ* deletion plasmid	This study
pCVD442-Δ*envZ*Δ*ompR*	*envZ-ompR* deletion plasmid	This study

**Table 2.** Primers used in this study

Primers	Sequence (5′-3′)				
For Construction					
pCVD442-fwd			GGCTGTCAGACCAAGTTTACTCATATATACTTTAGATTG		
pCVD442-rev			GCAGATACTCTTCCTTTTTCAATATTATTGAAGCATTTATCAGGGTTATTG		
Δ*dnaK*-A			GAAAAAGGAAGAGTATCTGCGGTACGGTACGGCCATACTGTTCG		
Δ*dnaK*-B			GCGATTAACCCATCTAAACGTCTCCACTAAAAAATCGTCATC		
Δ*dnaK*-C			CGTTTAGATGGGTTAATCGCCCTGATGCAGGGTAGATAAC		
Δ*dnaK*-D			GATTAATTGTCAAGGCTAGCGTGCTGTTTCACCTGCACCTGAAC		
Δ*sidA-*A			GAAAAAGGAAGAGTATCTGCGAACCACGCTTTTGCGTAGTATTAAC		
Δ*sidA-*B			CAAATCAGTCCAATGTCACTTTCCTTCATGCTGTG		
Δ*sidA-*C			GTGACATTGGACTGATTTGAGCGGGTCATTTGTTC		
Δ*sidA-*D			GATTAATTGTCAAGGCTAGCATCTTCAAGTATGCGTCGTATC		
Δ*hmsP*-A			GAAAAAGGAAGAGTATCTGCGTGGATGAACGGCTATTACTAC		
Δ*hmsP*-B			CGAAACCATCGCCATCTGTTTTATCGTGAGGGAAC		
Δ*hmsP*-C			AGATGGCGATGGTTTCGGCCGTTTCCCCGTGACGTCGCTGCAAAAGGTGTAAG		
Δ*hmsP*-D			GATTAATTGTCAAGGCTAGCTACCGATGGTGAACGCCATC		
Δ*recA-*A			GAAAAAGGAAGAGTATCTGCGTTGGCGATCACACCGAAGCAAG		
Δ*recA-*B			TCTTCGTTGGTCTGCTTGTTTTCGTCGATAGCCATTTTTAC		
Δ*recA-*C			ACAAGCAGACCAACGAAGAGTTTTAATCTCAAG		
Δ*recA-*D			GATTAATTGTCAAGGCTAGCCACGTCCTTGAACTGGTTCATC		
Δ*rpfR-*A			GAAAAAGGAAGAGTATCTGCGATGGTGGGTCAACAATCAATG		
Δ*rpfR-*B			AGGCATTCGTCGTCATCACAACC		
Δ*rpfR-*C			GTGATGACGACGAATGCCTGAGCTGCAATCACGTC		
Δ*rpfR-*D			GATTAATTGTCAAGGCTAGCAGCAGCTTCAGGCACGCAAG		
Δ*nlpD*-A			GAAAAAGGAAGAGTATCTGCGTTCTGAATCAACTGCGTGCTCAGG		
Δ*nlpD*-B			GCACTCTGCCAATTTATCGCTGCGATGGCGGCATAATCATAATCATCC		
Δ*nlpD*-C			CGATAAATTGGCAGAGTGCGCTTCTTCAG		
Δ*nlpD*-D			GATTAATTGTCAAGGCTAGCGCAGACCGAAACGACGGGCCAGAAC		
Δ*envZ-*A			GAAAAAGGAAGAGTATCTGCGGTTTTGTGATGAAAAGTGAAG		
Δ*envZ-*B			CTAGCTGTTCGAGAAGCGCAGCTTCCTCATG		
Δ*envZ-*C			GCTTCTCGAACAGCTAGTTTTCTGTTCACGCCATC		
Δ*envZ-*D			GATTAATTGTCAAGGCTAGCGATGACGGCGTATTTAACTTC		
Δ*envZ*Δ*ompR-*A			GAAAAAGGAAGAGTATCTGCGTCATTCATCATGGCAATATCATC		
Δ*envZ*Δ*ompR-*B			CCGTAGGCTCAAACAGTTGTAACGCCATTC		
Δ*envZ*Δ*ompR-*C			AACTGTTTGAGCCTACGGTAGTTAAAAACAGCTAGTTTTCTG		
Δ*envZ*Δ*ompR-*D			GATTAATTGTCAAGGCTAGCCCGAACCGGATATCTATCATG		
*nlpD*-comp-fwd			GGTCTAGATAGAGAAGTAAACCATTCCAG		
*nlpD*-comp-fwd			GGTCTAGATCTTCATTTAAGTCATGAAC		
*nlpD*(his)-fwd			GGTCTAGATAGAGAAGTAAACCATTCCAG		
*nlpD*(his)-rev			GGTCTAGATTAATGATGATGATGATGATGTCGCTGCGGCAAATAGC		
For sequencing confirmation					
Δ*dnaK*-E		GTGGCACGACGTCGGGTAAATC			
Δ*dnaK*-F		GTGGCCGCCATCGCAAAGTTGATC			
Δ*sidA*-E		GTGGTGCAGACCGGAGAAGTGGTC			
Δ*sidA*-F		GTGGCACATCCACCGGGTGAATGCG			
Δ*hmsP*-E		GTGGTCTTAACGCCGATCAGCTCAC			
Δ*hmsP*-F		GTAGAAGCAGACAATCAGCTG			
Δ*recA-*E		GTGGAATACAGCGTCGGCCAGCTG			
Δ*recA-*F		GTGGTTCCAGGTCGTTGTGCTTAC			
Δ*rpfR*-E		GTGGCTTCCAGTCGTTTGCGGAAATC			
Δ*rpfR-*F		GTGGATACAGCGCGTTTTACTCAAC			
Δ*nlpD-*E		GTGGAGTGAACGGGCAATGGTAAAC			
Δ*nlpD-*F		GTGGACATCTTCAAGCGTTGCC			
Δ*envZ-*E		GTGGTTGGCAGCGTCGTCATATCG			
Δ*envZ-*F		GTGGCACCGGCAAAACGACGCTTTC			
Δ*envZ*Δ*ompR*-E		GTGGATCAAGCAGGCGAATGGCAG			
Δ*envZ*Δ*ompR-*F		GTGGCACCGGCAAAACGACGCTTTC			
For RT-PCR					
*nlpD* RT-F *nlpD* RT-R *hmsP*-RT-F *hmsP*-RT-R *sdiA*-RT-F *sdiA*-RT-R *recA*-RT-F *recA*-RT-R *rpfR*-RT-F *rpfR*-RT-R 16s rRNA-F 16s rRNA-R		GTTTACAATCGCAAGTATG CCAGGCGATATAAAACAG CGATCACCTGGTGCATCAAC TGCAGACGCGGAATAGTGAG TATTTCGCGCTGTGCGTTCG TCCGCCTGGTAATGCTCAAG GGTTGATCTTGGCGTGAAGC GAGGAAGTTGGTCGCATTCG ATCGCCACGGCAACATTCAG CTTCAGCGGGCGTCATAAAG GCCTCATGCCATCAGATGTG TCTGGACCGTGTCTCAGTTC			

### Random mutagenesis and screening

The transposon mutagenesis in the strain *C. sakazakii* was performed according to protocols described previously [58]. Shortly, a transposon insertion library was constructed by using the EZ-Tn5< KAN-2> Tnp Transposome kit (NovoBiotec, China) according to the manufacturer’s instruction. To select for transposon insertion clones, 100 μl aliquots were plated onto LB agar plates containing 50 μg/ml kanamycin, and plates were incubated for 24 h at 37°C. Afterward, single colonies were rinsed three times with PBS. The bacterial suspension was centrifugated at 12,000 rcf for 1 minute. The supernatant was discarded, and the residue was washed twice with LB broth (pH 3.0). Aliquots (1 mL) were diluted 10-fold in LB broth (pH 3.0). After half an hour, 5-mL aliquots of the dilution were subcultured in 5 mL LB broth (pH 3.0) containing 100 mg/L gentamicin at 37°C with shaking for 1 hour. The strains were then serially diluted and plated on LB agar (pH 7.0). The colonies growing on LB agar were further tested in acidic challenge assays to determine their acid sensitivity. The randomness of insertions was verified according to the previously described method [59]. Genomic DNA was analyzed from individual mutants by Southern blotting using a digoxigenin probe against the kanamycin-resistance cassette contained within the transposon to confirm single transposon insertions.

### Bacterial survival assays

The bacterial survival assays were performed according to protocols described previously with small changes [60]. In the activation assays, 200 mM H2O2 and pH 3.0 were used.

### Macrophage survival assay

Macrophage survival was analyzed as described previously with minor changes [10], namely, that THP-1 cells were differentiated into macrophages using phorbol 12-myristate 13-acetate (Aladdin, China) (PMA) treatment at 60 ng/mL for 48 hours, and interferon-γ (ProteinTech, USA) (IFN-γ) was used to activate macrophages for enhanced microbial killing. *C. sakazakii* strains were added to THP-1 cells at an MOI of 100. *Salmonella enterica* serovar Typhimurium and DH5α at the same MOI were used as positive and negative controls, respectively.

### Intracellular pH measurement

Intracellular pH measurement assays were performed as described previously [61], except that the gene *pHluorin2*, which encodes an enhanced, ratiometric, pH-sensitive green fluorescent protein, was used in the pACYC184 vector and transformed it into *C. sakazakii*. Images were acquired using a Leica SPE confocal microscope (Leica, Germany) with dual excitation at 405 nm and 485 nm and an emission filter of 535 nm.

### RT-PCR

RT-PCR was performed in a Bio-Rad CFXConnect™ system (Bio-Rad, USA) using SYBR® green mix (NEB, USA) according to standard methods. The level of transcription was assessed through qPCR and normalized with internal control 16s rRNA using specific primers listed in Table 2. Thermocycling conditions for qPCR were as follows: initial denaturation at 92°C for 3 min and then denaturation at 92°C for 5 s, annealing at 56°C for 5 s, extension at 72°C for 5 s, and melt curve analysis at 65–95°C. Amplification of the single PCR product was confirmed by monitoring the dissociation curve followed by melting curve analysis.

### Rat virulence assay

Rat infections were conducted as described previously with minor changes [62]. *Salmonella enterica* serovar Typhimurium and *E. coli* DH5α served as positive and negative controls, respectively. Uninfected group was treated with a single dose of LB (100 μL) and infected groups with bacterial culture (10^7^ CFU) by oral gavage. Then, the animals were maintained atypical condition with mother. The survival of the infected animals was recorded at defined time intervals. To analyze bacterial colonization of organs, the rats were sacrificed 24 hours after infection. Organs were homogenized in PBS, the homogenates were serially diluted, and the number of bacteria was determined by plating the dilutions on LB agar.

### Adhesion assay

Bacterial adhesion was measured as described previously with small modifications [63]. C. sakazakii was prepared and applied to the Caco-2 cell monolayer at an MOI of 100. After 45 min incubation, the plates were washed three times with PBS, and the cells were lysed in 1% Triton X-100 (Abcone, China). The cell suspensions were serially diluted and plated on LB agar for enumeration of adherent bacteria. *Salmonella enterica* serovar Typhimurium was included as a positive control for adhesion to Caco-2. *Listeria monocytogenes* EGD-e was used as a positive control for adhesion to HBMECs. *E. coli* DH5α served as a negative control for adhesion to both Caco-2 and HBMECs.

### Immunoblot analysis of His6-tagged proteins

Immunoblot analysis was performed as described previously with small modifications [31]. Briefly, bacterial samples were disrupted by sonication (TissueLyser, China). The cell lysates were subsequently centrifuged, and the precipitates were transferred to membrane protein dissolution buffer (Sangon Biotech). The dissolved samples were used in western blotting. Protein concentration was determined using a BCA protein assay kit (Abcam, China). The proteins were transferred to nitrocellulose membranes (Bio-Rad, USA), and the membranes were blocked with 3% (w/v) milk (BD, USA) solution before incubation with monoclonal HRP-conjugated anti-6× His antibodies (Abcam, UK) diluted in 5% (w/v) BSA (Sangon Biotech). Protein signals were detected by HRP Substrate (Bio-Rad, USA) and Enhanced ECL Chemiluminescent Substrate (MKBio, China).

### Propidium iodide (PI) assay

The PI assay was performed as described previously with small modifications [58]. Briefly, *C. sakazakii* were grown in LB broth up to the mid logarithmic phase, harvested, washed, and adjusted to 10^6^ CFU/ml in LB at pH 3.0 or in LB at pH 7.0. The cells were incubated at 37°C for 2 hours. Subsequently, the cells were washed in PBS buffer and incubated with PI (1.3 μg/ml) at 37°C for 20 min in dark. A total of 10,000 cells were acquired for each flow cytometry analysis using a flow cytometer (Becton Dickinson).

### Ethics statement

The study was approved by the Ethics Committee of our department, and written informed consent was obtained from all participants before the study.

### Statistical analysis

Statistical analysis of all data was conducted using the GraphPad Prism program GraphPad (version 8.3). Significant differences were identified using Student’s two-tailed unpaired t-test (*, P < 0.05; **, P < 0.01; ***, P < 0.001, ns, not significant).

## Supplementary Material

Supplemental Material
