# Supplementary figures and images for: The lipoprotein NlpD in Cronobacter sakazakii responds to acid stress and regulates macrophage resistance and virulence by maintaining membrane integrity: Running Title: Identification and characterization of a novel factor involved in acid tolerance and virulence in Cronobacter sakazakii
Source: Virulence. 2021 Jan 18;12(1):415–29. doi: 10.1080/21505594.2020.1870336 (PMC7834084; doi:10.1080/21505594.2020.1870336)

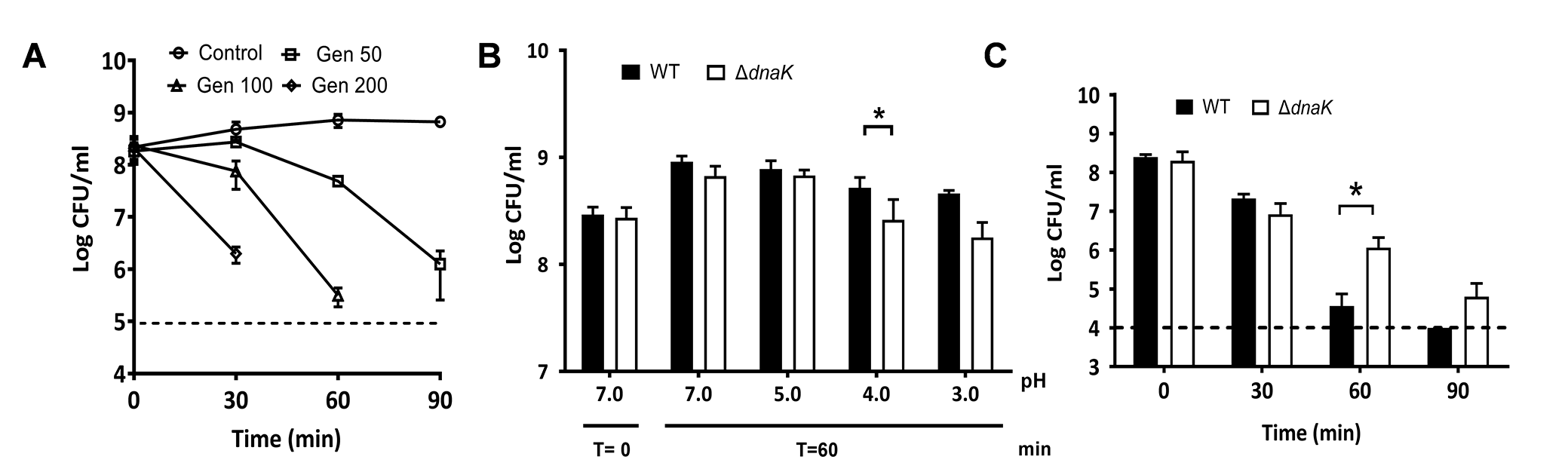

Supplement: Supplemental Material [file KVIR_A_1870336_SM7407.zip › SUPPLEMENTARY/Fig S1.tif]

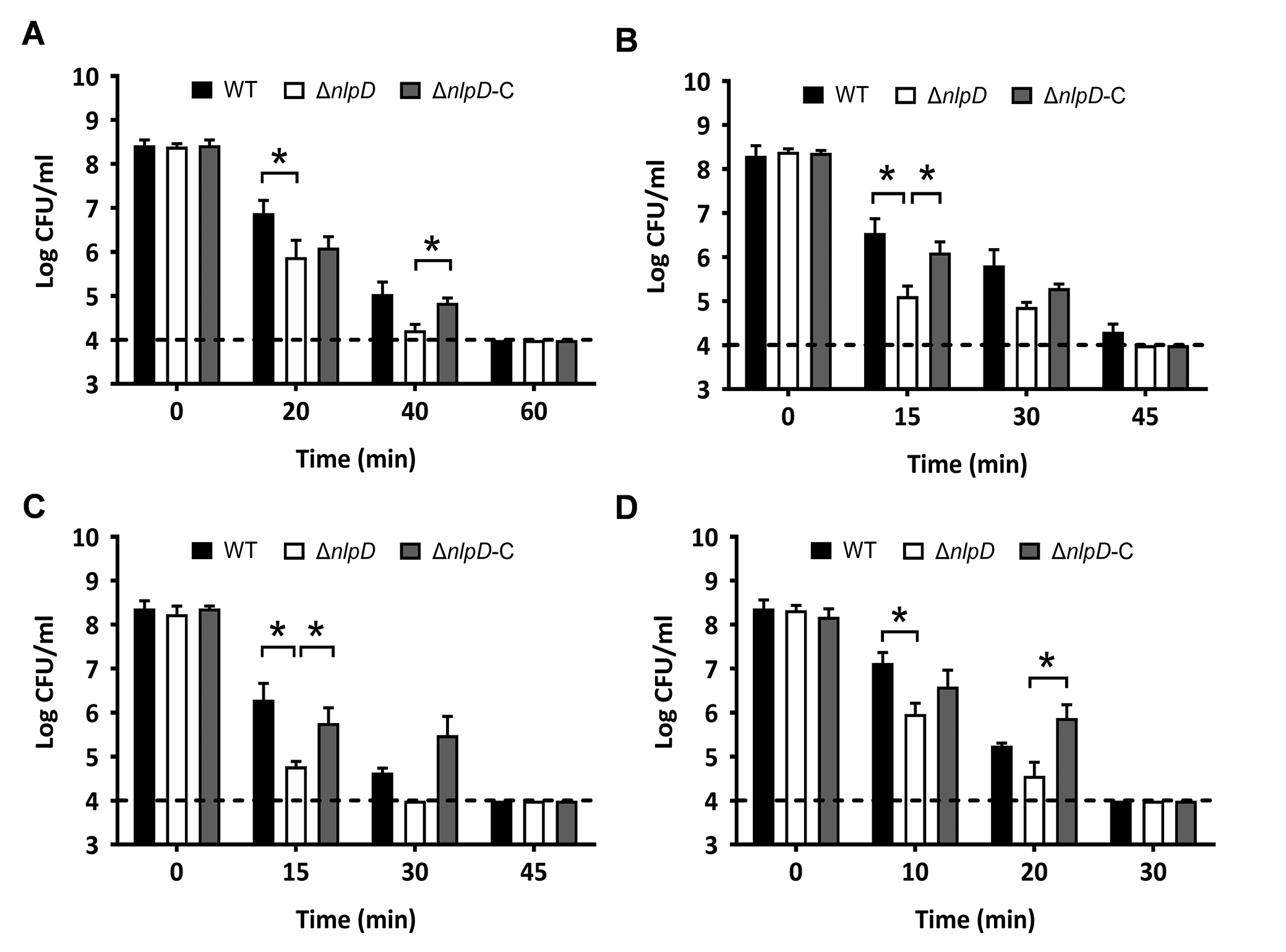

Supplement: Supplemental Material [file KVIR_A_1870336_SM7407.zip › SUPPLEMENTARY/Fig S2.tif]

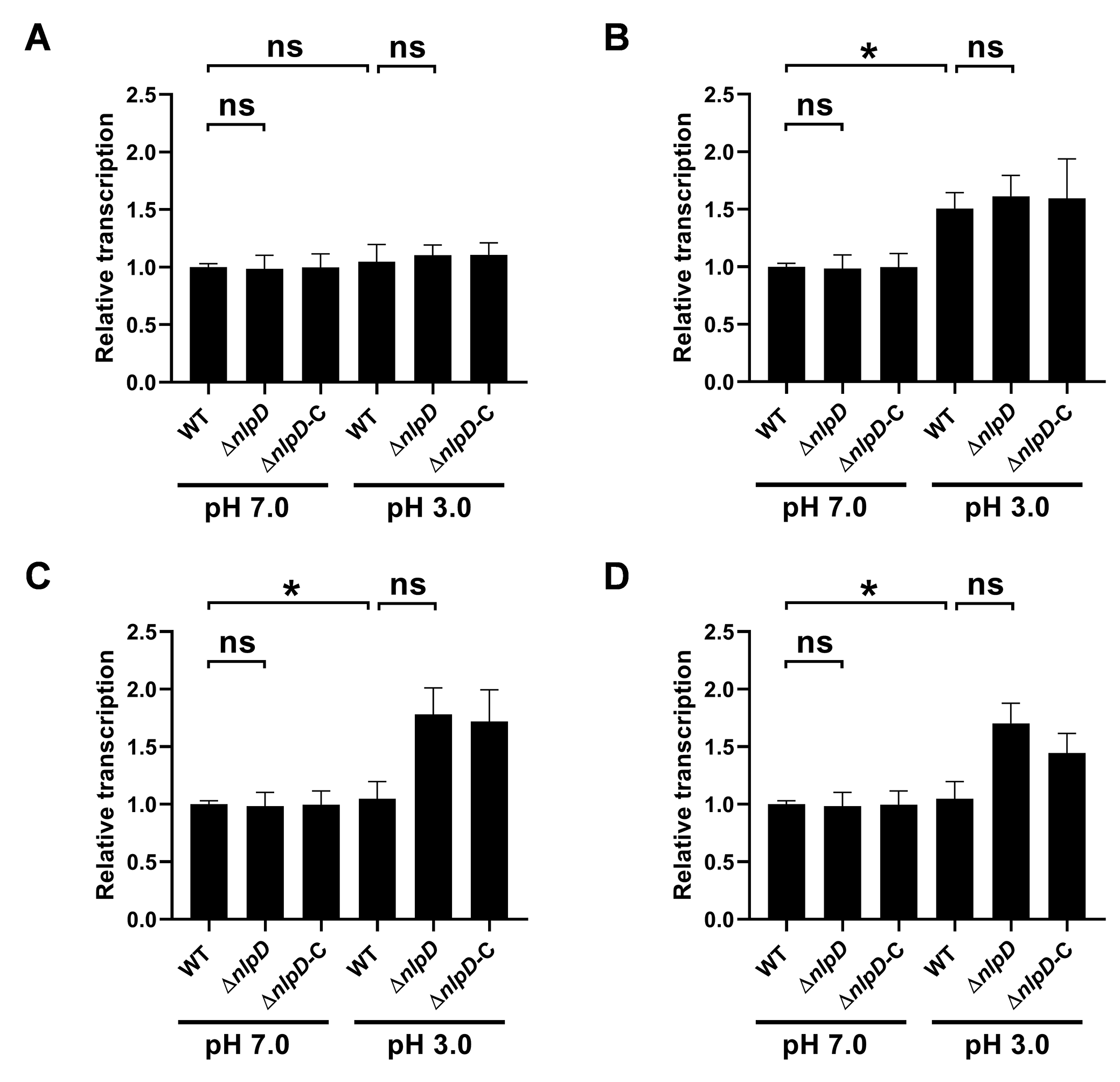

Supplement: Supplemental Material [file KVIR_A_1870336_SM7407.zip › SUPPLEMENTARY/Fig S3.tif]
